# Atrial Flutter Masquerading as an ST-segment–elevation Myocardial Infarction in a Patient with Dextrocardia

**DOI:** 10.19102/icrm.2022.130701

**Published:** 2022-07-15

**Authors:** Cody Carter, Ankur N. Shah, Mitchell Stelzer, Asim Ahmed

**Affiliations:** ^1^Department of Internal Medicine, Doctors Hospital, Columbus, OH, USA; ^2^Department of Electrophysiology, Ascension Sacred Heart, Pensacola, FL, USA; ^3^Department of Cardiology, Doctors Hospital, Columbus, OH, USA; ^4^Department of Cardiology, St. Vincent Hospital, Indianapolis, IN, USA

**Keywords:** Atrial flutter, catheter ablation, congenital heart disease, intracardiac electrophysiology, mapping

## Abstract

Electrocardiogram (ECG) findings suggestive of an ST-segment–elevation myocardial infarction (STEMI) often lead to emergent left heart catheterization. Occasionally, non-coronary conditions mimic ECG findings of STEMI, resulting in an increased risk and expenses from emergent transportation and procedures. In this report, we describe diagnostic and management strategies for a case of 1:1 atrial flutter in a patient with dextrocardia presenting as a STEMI.

## Introduction

Electrocardiogram (ECG) findings suggestive of an ST-segment–elevation myocardial infarction (STEMI) often lead to emergent left heart catheterization (LHC). Occasionally, non-coronary conditions mimic ECG findings of a STEMI, resulting in an increased risk and expenses from emergent transportation and procedures. While the overall incidence has been declining over the last decade given the advances in guideline-directed medical therapy, this still accounts for a substantial amount of emergency department visits.^1^ Between 50%–85% of patients presenting with chest pain who meet the criteria for STEMI were found to have a diagnosis other than acute coronary syndrome.^2,3^ Here, we report a case and discuss the management of 1:1 atrial flutter in a patient with dextrocardia presenting as a STEMI.

## Case presentation

**Figure 1:**
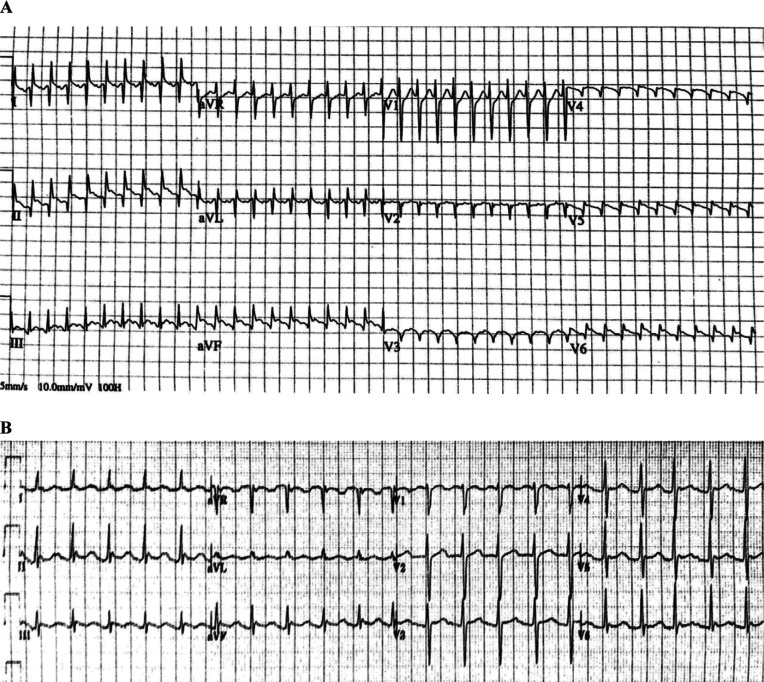
**A:** Narrow complex tachycardia with deep Q-waves across the precordium from leads V2–V6, I, II, and aVL along with inferolateral ST-segment elevation, raising the possibility of a large transmural infarct. **B:** After adenosine and lead reversal, the Q-waves previously seen in leads I, II, aVL, and V2–V6 had disappeared, further supporting the diagnosis of atrial flutter.

**Figure 2:**
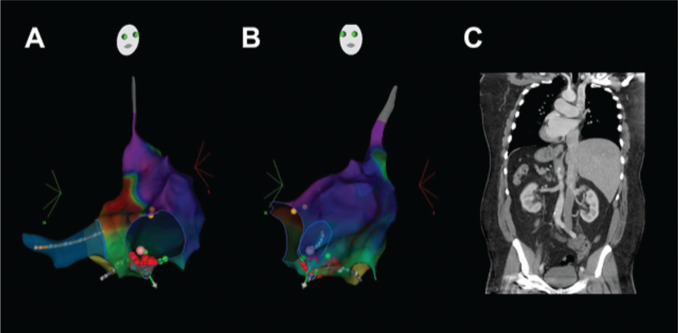
A right anterior oblique projection **(A)** and a left anterior oblique projection **(B)** are shown. Activation maps in **A** and **B** illustrate counterclockwise atrial flutter, with red and purple interfaces exhibiting the early and late components of the re-entry circuit, respectively. **C:** Computed tomography imaging confirming dextrocardia with situs inversus in addition to continuity of the inferior vena cava in the right atrium is depicted.

**Figure 3:**
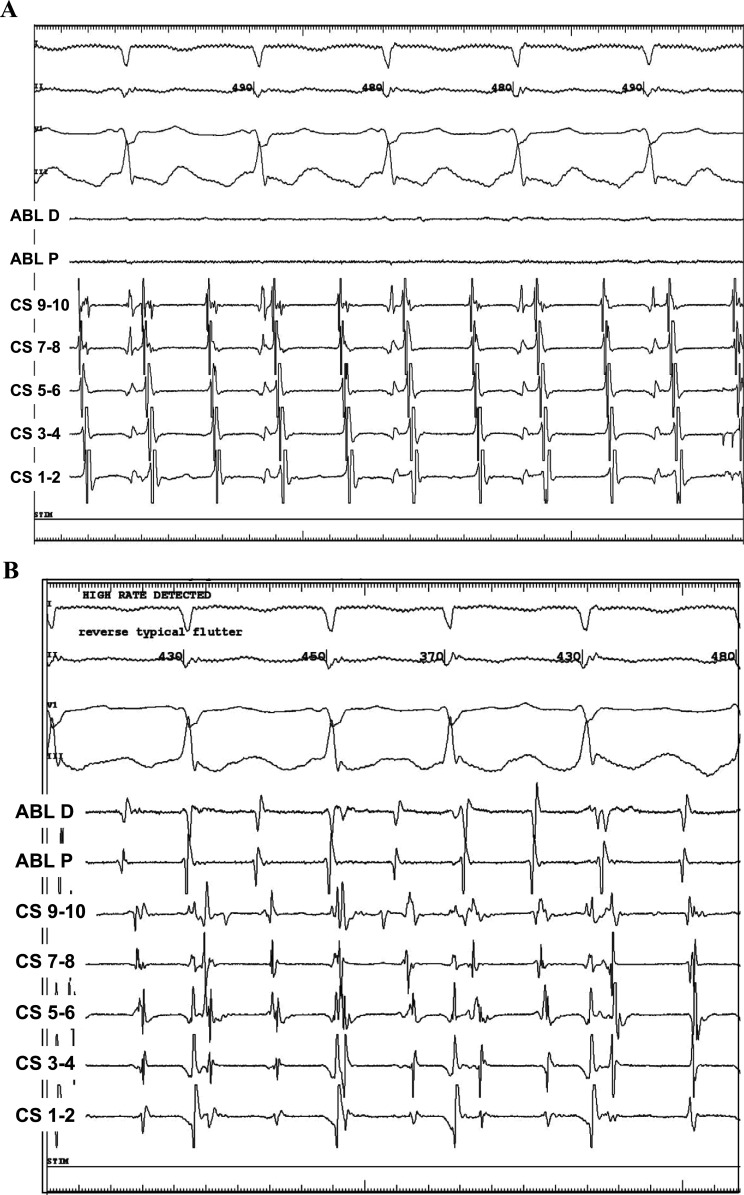
**A:** The precordial leads were reversed, and the entrainment attempt terminated the tachycardia as the patient was under anesthesia, but atrial ectopy caused atrial fibrillation, which organized into multiple types of flutter after a few minutes. Clockwise cavotricuspid isthmus (CTI) flutter was confirmed via activation mapping. **B:** With precordial leads still reversed, counterclockwise CTI flutter was also confirmed via activation mapping.

A 63-year-old man presented to an outside emergency room for acute chest pain and shortness of breath. An ECG revealed a narrow complex tachycardia at 240 bpm with an inferolateral ST-segment elevation and deep Q-waves across the precordium and leads I, II, and aVL **(Figure 1A)**. A STEMI alert was called, and the patient was transferred to our cardiac catheterization laboratory. Upon arrival, the patient exhibited hypotension, chest pain, and dyspnea. In order to either slow down or terminate the atrial tachyarrhythmia and improve diastolic filling, 12 mg of adenosine was administered intravenously, which resulted in the slowing down of the ventricular rate and the unmasking of flutter waves. Although hemodynamics improved with a reduction in heart rate, the patient continued to exhibit mild hypotension. Due to the persistence of chest pain and dyspnea in addition to precordial Q-waves concerning for a large transmural infarct, coronary angiography was performed and revealed normal coronaries in addition to probable dextrocardia, suggesting this was the likely etiology of the Q waves. Following LHC, a repeat ECG was performed with a reversal of both limb and precordial leads, confirming the resolution of Q-waves **(Figure 1B)**. Rate-control options were limited due to relative hypotension, and the patient had early recurrence of atrial flutter after cardioversion despite the initiation of anti-arrhythmic therapy. Following computed tomography (CT) imaging, which confirmed dextrocardia with situs inversus and inferior vena cava continuity into the right atrium **(Figure 2C)**, the patient underwent a fluoroless electrophysiology study and radiofrequency ablation under 3-dimensional electroanatomic and intracardiac echo guidance **(Figures 2A and 2B)**. The electrophysiology study identified both typical (in this case, clockwise) flutter **(Figure 3A)** and subsequently reverse typical (counterclockwise) flutter **(Figure 3B)**. Radiofrequency ablation along the cavotricuspid isthmus terminated the flutter with subsequently confirmed bi-directional block. Following ablation, 30-day event monitoring was performed to assess for underlying atrial fibrillation. He exhibited a <1% atrial fibrillation burden and otherwise remained free of atrial flutter.

## Discussion

While dextrocardia and limb lead reversal both exhibit (1) an inverted lead I, (2) reversal of aVR and aVL, and (3) reversal of leads II and III, dextrocardia differentiates itself by having poor R-wave progression in the precordial leads. In the initial ECG **(Figure 1)**, ST-segment elevations noted along II and aVF with Q-waves throughout I, II, aVF, aVL, and the precordium were concerning for a transmural infarct. After angiography showed no obstructive coronary disease, adenosine slowed down the tachycardia, revealing the underlying atrial flutter. This proved that the ST-segment elevations were exclusively due to the underlying flutter waves, which was further supported during the reversal of the chest leads **(Figure 1B)**.

STEMI in patients with dextrocardia is an extremely rare finding, with <100 cases reported worldwide. Unrecognized atrial flutter can mimic Q-waves in the setting of dextrocardia. In our case, adenosine was a valuable tool in revealing the underlying arrhythmia. If dextrocardia is suspected, ECGs obtained with limb and precordial lead reversal can aid in the diagnosis. Regarding management, multimodality imaging is particularly valuable in guiding therapeutic options and choosing viable approaches. Once the tachyarrhythmia is confirmed, imaging should be performed to assess for other associated congenital cardiac abnormalities prior to performing an intervention.

Dextrocardia is a rare condition with an estimated incidence of 1 per 12,000 live births, 30% of these being associated with situs inversus.^4^ It is important to differentiate this from dextroversion (dextrocardia with situs solitus), in which the ventricles are shifted to the right with the atria remaining in their usual position. Dextroversion has a 90% incidence of additional cardiac abnormalities, including anomalous pulmonary venous return, tetralogy of Fallot, septal defects, coarctation of the aorta, and transposition of the great arteries, whereas dextrocardia with situs inversus has a much lower incidence of concomitant heart defects.^5,6^ Thus far, there has been no association with dextrocardia and coronary artery disease. There are a few case reports of associated arrhythmias; however, these were all accompanied by other congenital abnormalities, such as persistent left superior vena cava (PLSVC) draining into the coronary sinus.^7–9^ It is theorized that these arrhythmias are caused by structural changes in the conduction system due to the proximity of anatomic insertions of the PLSVC. This leads to dilation of the coronary ostium and alterations in cardiac anisotropy. While this abnormality is usually diagnosed incidentally, our patient did not have a PLSVC and was not at an increased risk of developing macro-reentrant arrhythmias compared to the general population.

## Conclusion

Patients with dextrocardia and 1:1 atrial flutter may present as having STEMI due to flutter waves coinciding with ST segments and Q-waves. Repeat ECG with slower atrioventricular conduction during flutter may reveal the diagnosis. After the diagnosis is made, CT imaging should be used to confirm inferior vena cava continuity to guide vascular access for procedural intervention.
